# ADMET-Guided Docking and GROMACS Molecular Dynamics of *Ziziphus lotus* Phytochemicals Uncover Mutation-Agnostic Allosteric Stabilisers of the KRAS Switch-I/II Groove

**DOI:** 10.3390/ph18081110

**Published:** 2025-07-25

**Authors:** Abdessadek Rahimi, Oussama Khibech, Abdessamad Benabbou, Mohammed Merzouki, Mohamed Bouhrim, Mohammed Al-Zharani, Fahd A. Nasr, Ashraf Ahmed Qurtam, Said Abadi, Allal Challioui, Mostafa Mimouni, Maarouf Elbekay

**Affiliations:** 1 Laboratory of Applied Chemistry and Environment (LCAE), Faculty of Science, University Mohammed Premier, Bd. Med VI B.P. 717, Oujda 60000, Morocco; abdessadek.rahimi@ump.ac.ma (A.R.); abdessamad.benabbou@ump.ac.ma (A.B.); moh.merzouki@gmail.com (M.M.); saidabma@yahoo.fr (S.A.); a.challioui@ump.ac.ma (A.C.); mimouniosrn@gmail.com (M.M.); e.maarouf@ump.ac.ma (M.E.); 2 Laboratoires TBC, Laboratory of Pharmacology, Pharmacokinetics, and Clinical Pharmacy, Faculty of Pharmaceutical and Biological Sciences, 59000 Lille, France; mb@laboratoires-tbc.com; 3 Biology Department, College of Science, Imam Mohammad Ibn Saud Islamic University (IMSIU), Riyadh 11623, Saudi Arabia; mmyalzahrani@imamu.edu.sa (M.A.-Z.); aaqurtam@imamu.edu.sa (A.A.Q.)

**Keywords:** KRAS inhibition, *Ziziphus lotus*, hyperin, molecular docking, molecular dynamics simulation

## Abstract

**Background/Objectives:** Oncogenic KRAS drives ~30% of solid tumours, yet the only approved G12C-specific drugs benefit ≈ 13% of KRAS-mutant patients, leaving a major clinical gap. We sought mutation-agnostic natural ligands from Ziziphus lotus, whose stereochemically rich phenolics may overcome this limitation by occupying the SI/II (Switch I/Switch II) groove and locking KRAS in its inactive state. **Methods:** Phytochemical mining yielded five recurrent phenolics, such as (+)-catechin, hyperin, astragalin, eriodictyol, and the prenylated benzoate amorfrutin A, benchmarked against the covalent inhibitor sotorasib. An in silico cascade combined SI/II docking, multi-parameter ADME/T (Absorption, Distribution, Metabolism, Excretion, and Toxicity) filtering, and 100 ns explicit solvent molecular dynamics simulations. Pharmacokinetic modelling predicted oral absorption, Lipinski compliance, mutagenicity, and acute-toxicity class. **Results:** Hyperin and astragalin showed the strongest non-covalent affinities (−8.6 kcal mol^−1^) by forging quadridentate hydrogen-bond networks that bridge the P-loop (Asp30/Glu31) to the α3-loop cleft (Asp119/Ala146). Catechin (−8.5 kcal mol^−1^) balanced polar anchoring with entropic economy. ADME ranked amorfrutin A the highest for predicted oral absorption (93%) but highlighted lipophilic solubility limits; glycosylated flavonols breached Lipinski rules yet remained non-mutagenic with class-5 acute-toxicity liability. Molecular dynamics trajectories confirmed that hyperin clamps the SI/II groove, suppressing loop RMSF below 0.20 nm and maintaining backbone RMSD stability, whereas astragalin retains pocket residence with transient re-orientation. **Conclusions:** Hyperin emerges as a low-toxicity, mutation-agnostic scaffold that rigidifies inactive KRAS. Deglycosylation, nano-encapsulation, or soft fluorination could reconcile permeability with durable target engagement, advancing *Z. lotus* phenolics toward broad-spectrum KRAS therapeutics.

## 1. Introduction

For more than four decades, the RAS oncogene family and KRAS in particular have epitomised the paradox of being both biologically central and chemically elusive. KRAS mutations drive roughly one-third of all solid tumours [1,2], yet the protein’s shallow, highly polarised surface and picomolar-affinity nucleotide pocket long discouraged classical active-site drug design [3]. Only in 2021 did the covalent G12C-targeted inhibitor sotorasib reach the clinic, proving that “undruggable” does not mean “undruggable forever”; however, it benefits only ≈ 13% of patients harbouring KRAS mutations, leaving a major unmet clinical need and highlighting the structural richness of *Ziziphus lotus* phenolics as a promising alternative to the liability-prone covalent inhibitors currently in use [4,5]. Covalent G12C ligation also suffers intrinsic liabilities, including Michael acceptor toxicity, adaptive bypass via SHP2, or EGFR reactivation, and rapid on-target resistance through secondary substitutions or allele switching [6,7,8,9]. Consequently, attention has pivoted toward non-covalent allosteric pockets that, rather than hacking the nucleotide switch directly, modulate the protein’s dynamic control elements, most conspicuously the SI/II(Switch I/Switch II) groove that cradles the flexible Switch I and II loops [10]. Crystallographic campaigns and fragment screens have revealed that subtle chemical nudges in this lateral cleft can stiffen the loops enough to retain GDP and obstruct effector engagement, offering a mutation-agnostic path to KRAS silencing [9,11,12].

Fragment-derived scaffolds such as BI-2852 and EX185 supplied the first proof of concept but simultaneously exposed the liabilities of low-molecular-weight hydrophobes: sub-micromolar affinities arrived hand in hand with nanomolar solubility, metabolic lability, and broad off-target panels typical of lipophilic binders. Macrocycles and peptidomimetics subsequently broke the nanomolar barrier yet stumbled over permeability bottlenecks, synthetic complexity, and prohibitive cost of goods [10,12,13,14]. These setbacks have reinvigorated interest in the natural-product space, whose structural diversity, dense stereochemistry, and pre-organised pharmacophores frequently outperform synthetic libraries in three-dimensional complexity and ligand efficiency [15]. Flavonoids, stilbenoids, meroterpenes, and alkaloids in particular display polar–apolar amphiphilicity that resonates with the electrostatic mosaic of the SI/II floor, while epidemiological data furnish an encouraging baseline of human tolerability [16,17].

Turning such scaffolds into bona fide leads, however, demands reconciliation of four interlocking challenges [18]. First, plant-derived collections are often under-annotated, necessitating high-throughput virtual triage that couples docking with rigorous rescoring to weed out false positives from tautomeric ambiguity or pan-assay interference substructures. Second, the multidentate hydrogen-bond networks that award many natural compounds their high in silico stickiness frequently inflate polar surface area beyond Lipinski boundaries, depressing passive permeability; robust ADME-tox (Absorption, Distribution, Metabolism, Excretion, and Toxicity) filters and transporter modelling must, therefore, be integrated early to avoid affinity-permeability deadlocks [19,20]. Third, because loop breathing and β-sheet fraying dominate the microsecond landscape of KRAS, static docking snapshots provide, at best, a frozen caricature of binding; explicit solvent molecular dynamics, principal component analysis, and free-energy decomposition are indispensable for identifying ligands that reinforce the desirable “rigid-flexible-rigid” gradient without over-constraining the catalytic core [21,22]. Finally, formulation science looms large: polyhydroxylated hits may require pro-drug masking, nano-encapsulation, or targeted release in the tumour’s acidic microenvironment to realise their pharmacological promise [23,24].

The literature from the past five years underscores both promise and pitfalls. Structure-guided evolution of BI-2852 analogues delivered sub-100 nM binders that arrest RAS-SOS exchange and shrink KRAS-driven xenografts, but only under continuous intravenous infusion, highlighting the need for improved physicochemical properties [25]. Parallel fragment-merging efforts generated bicyclic hetero-aromatics that stabilise an “α-state” conformation, yet their mouse liver clearance exceeds 50 mL min^−1^ kg^−1^, jeopardising systemic exposure [26]. Deep-learning retrosynthesis and DNA-encoded libraries have broadened the chemical vocabulary of SI/II ligands, but hit validation still circles back to the classic bottlenecks of solubility, selectivity, and metabolic soft spots [27]. On the natural-product front, oxidised meroterpenoids from marine fungi and prenylated stilbenes from Macaranga species show mid-micromolar inhibition of KRAS-effector interactions in vitro [28]. Yet none has crossed the threshold of demonstrable pathway shutdown in KRAS-addicted cell lines, let alone in vivo anti-tumour activity, because permeability and metabolic liability remain unresolved [29,30].

The convergence of fragment-based design, advanced molecular-simulation techniques, and the rediscovery of natural-product chemical space thus constitutes fertile ground for the next generation of non-covalent, mutation-agnostic KRAS inhibitors. By articulating the mechanistic subtleties of SI/II modulation, acknowledging the physicochemical penalties of multidentate binding and embracing modern in silico triage married to iterative optimisation, the field is poised to turn an erstwhile “undruggable” liability into a tractable therapeutic opportunity fit for combination regimens targeting the broader MAPK pathway.

This study employs an integrated virtual workflow that starts with molecular docking into the SI/II groove of KRAS, applies ADMET filtering, and then runs an explicit solvent 100 ns molecular dynamics simulation to verify complex stability. Drawing on recent LC-HRMS and HPLC-DAD/MS surveys that catalogued more than 180 specialised metabolites in the ecologically abundant shrub *Ziziphus lotus,* together with its long-standing ethnomedicinal use against cancer, we prioritised five recurrent phenolic scaffolds (catechin, hyperin, astragalin, eriodictyol, and the prenylated phenyl-propionate amorfrutin A) for virtual screening [31]. Results highlighted hyperin as the most potent stabiliser, conferring marked rigidity to the SI/II pocket, while astragalin emerged as a backup lead amenable to optimisation through deglycosylation, nano-encapsulation, or soft fluorination, thereby paving the way for broad-spectrum RAS inhibitors.

## 2. Results and Discussion

### 2.1. ADME Results

#### 2.1.1. Physicochemical and SwissADME Bioavailability Comparison of Natural KRAS Candidates Versus AMG-510

Table 1 compiles the structural determinants that govern a molecule’s likelihood of traversing oral absorption barriers: Lipinski’s “rule of five” predicts that passive permeability falls sharply once a compound exceeds 5 hydrogen-bond donors, 10 acceptors, 500 g·mol^−1^, or LogP > 5, whereas Veber’s rule stresses the need for reasonable rigidity (<10 rotatable bonds) and moderate polarity (TPSA ≤ 140 Å^2^) to preserve trans-membrane flux. SwissADME distills these descriptors into a Bioavailability Score (ABS) in which values of 0.17, 0.55, and 0.85 correspond to progressively higher chances (≈10%, 55%, 85%) of achieving at least 10% oral bioavailability in rats [32,33].

Within this framework, catechin (M1) exemplifies a classic polyphenol compromise: a moderate mass (290 g·mol^−1^) and near-optimal rigidity, yet five phenolic groups that, while keeping TPSA ~ 110 Å^2^ and LogP 0.85 inside the ideal window, expose the molecule to P-gp efflux and rapid phase-II conjugation; its ABS of 0.55, therefore, indicates that absorption remains plausible in the absence of strong protein binding. Amorfrutin A (M2) pushes hydrophobicity to the permissible limit (LogP 4.37) through an isoprenoid chain and a phenethyl fragment; its low polarity (TPSA 66 Å^2^) and seven rotamers still below Veber’s cut-off translate into the maximal ABS (0.85), foreshadowing efficient gastrointestinal diffusion that partly offsets its expected low aqueous solubility. By contrast, adding a galactoside (hyperin, M3) or glucose (astragalin, M5) moiety simultaneously inflates weight, polarity (TPSA > 190 Å^2^), and hydrogen-bond counts: two Lipinski violations and a catastrophic ABS (0.17) forecast virtually no epithelial passage unless vectorization or deglycosylation strategies are deployed. Eriodictyol (M4) sits between these extremes: its mass (288 g·mol^−1^), LogP 1.45, and TPSA 107 Å^2^ fall squarely within the ideal ellipse, as reflected by an ABS of 0.55; its low flexibility (a single rotatable bond) hints at greater metabolic stability than M1 while preserving good solubility.

The benchmark AMG-510 deliberately exceeds the mass limit (560 g·mol^−1^) yet embodies a “beyond-Ro5” design: through judicious placement of heteroatoms (HBA = 8; HBD = 1), two metabolically stabilising fluorines, and a rigid bicyclic core, it maintains a TPSA of ≈104 Å^2^ and LogP 4.05 within the absorption corridor, yielding an ABS identical to M1 and M4 despite its overweight. Overall, the probability of systemic exposure can be ranked as follows: amorfrutin A > eriodictyol ≈ catechin ≳ AMG-510 ≫ hyperin ≈ astragalin. Because KRAS-targeted anticancer efficacy ultimately depends on intracellular concentration, M2 and M4 emerge as the preferred prototypes, whereas M1 could benefit from methylated or fluorinated derivatives to curb conjugation, and M3/M5 must be converted to pro-aglycones or encapsulated in lipid nanocarriers if they are to pharmacokinetically rival the reference compound AMG-510.

**Figure 1:**
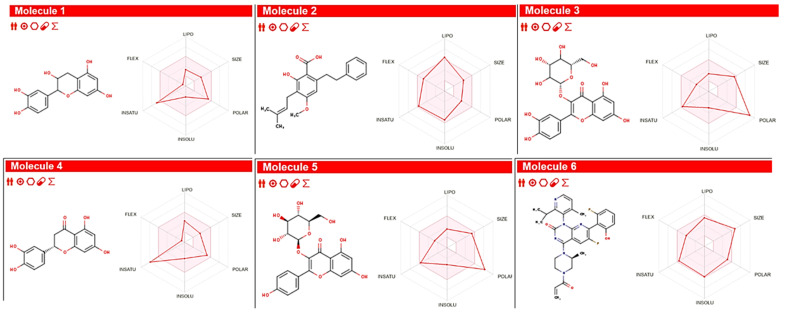
SwissADME bioavailability radar plots illustrating the six-parameter oral-drug-likeness profile (LIPO, SIZE, POLAR, INSOLU, FLEX, INSATU) of each natural compound (M1–M5) compared with AMG-510 (M6).

The bioavailability radars reveal (Figure 1), at a glance, how each scaffold balances the six physicochemical vectors that dictate oral drug-likeness. For catechin (M1) the profile is compact except for a pronounced POLAR spike, driven by its five phenolic hydroxyls that raise TPSA to the upper edge of the optimal zone; the same hydrogen-bond network depresses LIPO and lends excellent aqueous SOLU, while the single aliphatic bridge keeps FLEX and SIZE comfortably inside the pink area, structurally explaining why conjugation and P-gp efflux, rather than permeability, are its main liabilities [34]. Amorfrutin A (M2) shows the mirror image: an elongated LIPO and INSOLU cone that breaches the ideal region, the inevitable consequence of a phenethyl tail and an isoprenoid side chain that push cLogP beyond 4; yet its small aromatic core restrains SIZE, the carboxylate and methoxy temper POLAR, and seven rotors widen the FLEX petal without exceeding the permitted radius, together predicting good passive uptake if formulation overcomes low solubility. Hyperin (M3) produces the most distorted radar SIZE and POLAR fan, far outside the sweet spot because the galactose enlarges molecular weight and adds a forest of donors and acceptors, while the sugar’s sp^3^ enrichment contracts the INSATU wedge; although LIPO stays well within limits, the combined imbalance anticipates negligible passive permeation. A similar but slightly less extreme shape appears for astragalin (M5), where a glucose rather than galactose lowers mass and polarity just enough to shrink the overshoot, but the radar still confirms the need for deglycosylation or carrier systems. Eriodictyol (M4) traces an almost ideal hexagon: moderate lipophilicity from its flavanone ketone, a TPSA of ~107 Å^2^ from four hydroxyls, minimal rotatable bonds, and a largely aromatic skeleton that modestly narrows the INSATU sector, collectively suggesting a naturally well-balanced oral candidate. Finally, the reference AMG-510 (M6) illustrates a deliberate “beyond-rule-of-five” design: the SIZE petal touches the outer boundary owing to a 560 Da frame, but judicious heteroatom placement maintains POLAR near the center, bifluorination and a bicyclic core keep LIPO within range, and six rotors generate a FLEX domain that is broad yet tolerated; the overall symmetric radar signals that, despite its heft, AMG-510 preserves the physicochemical harmony required for sufficient gastrointestinal absorption and intracellular delivery to KRAS.

#### 2.1.2. In Silico ADME Profile and Interaction Risks (pkCSM Analysis)

PKCSM highlights how subtle architectural nuances dictate each compound’s in vivo fate (Table 2). Amorfrutin A (M2) attains the highest predicted human intestinal absorption (93%) thanks to its lipophilic phenethyl-isoprenoid tail and modest polar surface (TPSA 66 Å^2^), whereas the bulky, highly glycosylated scaffolds of hyperin (M3) and astragalin (M5) push TPSA beyond 190 Å^2^ and inflate hydrogen-bond counts, restricting uptake to 35% and 42%, respectively; catechin (M1) and eriodictyol (M4) occupy an intermediate window (72–79%), consistent with their balanced LogP and polarity, while AMG-510 (M6) achieves a remarkable 89% despite its mass by distributing heteroatoms to keep TPSA ≈ 104 Å^2^ and incorporating two fluorines that sharpen lipophilic permeability. Blood–brain barrier indices (logBB) confirm that only M2 (−0.26) approaches the −1 cut-off for cerebral access, whereas all other naturals and AMG-510 (−1.48) remain below the threshold, minimising off-target CNS exposure. A stricter CNS logPS filter (≈−2) reinforces this trend, with hyperin registering the poorest penetrance (−5.13). None of the flavonoids inhibit CYP2D6/3A4, but the nitrogen-rich, aromatic core of AMG-510 is predicted to block CYP3A4, foreshadowing drug–drug interaction risks and first-pass metabolic saturation [35,36]. The same cationic heteroaromatic motif renders AMG-510 an OCT2 substrate, a liability shared with many nephrotoxic agents. Vigilant renal monitoring or structural dampening of basicity (e.g., N-oxide pro-drug) could mitigate tubular accumulation [37]. To rescue weak profiles, deglycosylation or lipophilic ester pro-drugs of M3/M5 would collapse TPSA, boosting absorption and BBB metrics; nano-emulsions or self-micro-emulsifying systems can offset M2’s limited aqueous solubility without impairing its favourable clearance; strategic O-methylation/fluorination of catechin may temper conjugation and efflux; finally, CYP3A4 inhibition by AMG-510 could be alleviated via peripheral alkyl shortening or by co-administering non-competitive metabolic spare-capacity modulators, provided OCT2 engagement is simultaneously reduced through neutral bio-isosteric substitutions [38]. Altogether, these insights rationalise how each structural motif translates into distinct ADME snapshots and guide targeted chemical or formulation tweaks to converge in an optimised, KRAS-focused therapeutic profile.

#### 2.1.3. Integrated Thermal-Pharmacokinetic Profiling and Structure-Guided Optimisation Strategy

**Figure 2:**
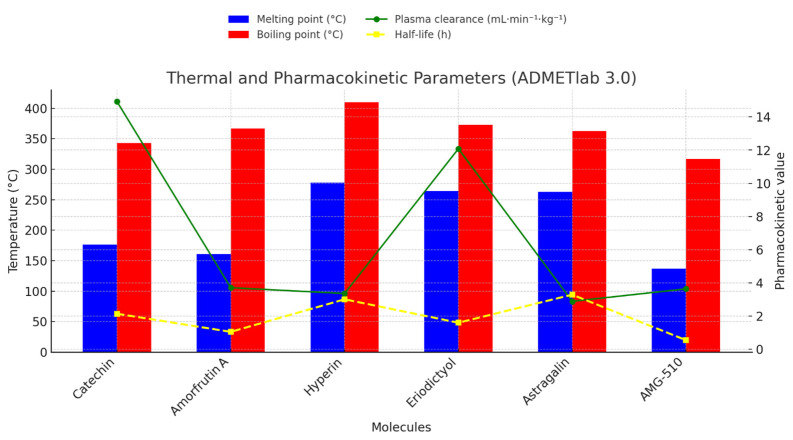
Comparative thermal (mp, bp) and pharmacokinetic (plasma clearance, t½) parameters of the five natural KRAS-targeting candidates versus the reference inhibitor AMG-510.

Based on the diagram in Figure 2, the combined thermal (mp, bp) and pharmacokinetic (Cl_plasma_, t_½_) fingerprints convert the underlying chemotypes of the six molecules into a coherent, measurable performance profile. Catechin, a compact trihydroxylated flavan-3-ol, melts at 176 °C and boils at 343 °C driven by an intramolecular H-bond network that stabilises the crystal lattice without excessive molecular mass; however, the same five phenolic hydroxyls render it highly susceptible to UGT/SULT conjugation, explaining the fast hepatic/plasma clearance (Cl_plasma_ ≈ 15 mL · min^−1^ · kg^−1^) and a modest half-life of 2.1 h [39]. Selective O-methylation or fluorination of one or two phenols or delivery as an ester pro-drug that regenerates the parent in tumour tissue would cap conjugation sites and slow clearance without eroding activity. Amorfrutin A replaces catechin’s polyphenolic core with a benzoic acid tethered to a phenethyl and isoprenoid tail; the dilution of hydrogen-bond donors lowers the melting point to 161 °C, yet dispersion forces along the C_10_ chain push the boiling point to 367 °C. Fewer polar handles (HBD = 2) reduce metabolic turnover, so Cl_plasma_ collapses to 3.7 mL · min^−1^ · kg^−1^ and t_½_ shrinks to 1 h, indicating rapid distribution but slower enzymatic removal; a self-emulsifying drug-delivery system (SEDDS) or insertion of a polar heteroatom in the aliphatic chain could raise aqueous solubility and extend systemic exposure. Hyperin and astragalin, quercetin- and kaempferol-3-O-glycosides, respectively, exhibit the highest thermal constants (mp ≈ 278/263 °C; bp ≈ 410/363 °C): the glycone massively expands hydrogen-bonding and lattice enthalpy [40,41,42]. Paradoxically, their clearances are low (≈3 mL · min^−1^ · kg^−1^) not because they evade metabolism but because the bulky, highly polar sugar limits passive diffusion into hepatocytes; the resulting volumes of distribution and slow hydrolytic deglycosylation lengthen half-lives to ≈3 h. Removing the sugar (pro-aglycone approach), acylating it transiently, or encapsulating the intact glycoside in lipid nanoparticles would compress TPSA, restore permeability, and still exploit in situ enzymatic release. Eriodictyol, a tetra-hydroxylated flavanone lacking a sugar, balances the two extremes: extensive π-π stacking lifts mp/bp to 264/373 °C, four phenols drive Cl_plasma_ upward (12 mL · min^−1^ · kg^−1^), and the half-life contracts to 1.6 h; introduction of a metabolically blocking methoxy group at C-7 or formulation in long-circulating micelles should damp clearance and lengthen residence time. The reference inhibitor AMG-510, engineered for covalent KRAS engagement, possesses a heteroaromatic, difluorinated scaffold with only one H-bond donor; weak crystal packing and lower polarity explain the lowest mp (137 °C) and bp (317 °C) in the series. Strategic halogenation shields labile sites, so metabolic extraction remains moderate (Cl_plasma_ ≈ 3.6 mL · min^−1^ · kg^−1^), yet irreversible cysteine binding in vivo shortens the apparent systemic half-life to 0.55 h; a controlled-release formulation or reversible docking “soft-covalent” analogue could prolong systemic exposure without sacrificing on-target residence. Overall, the data show a continuum from high-melting, low-clearance glycosides that linger in plasma but struggle to permeate cells to low-melting, rapidly cleared AMG-510, optimised for fast intracellular delivery; targeted chemical edits or advanced formulations can shift each candidate toward the optimal middle ground of thermal robustness, permeability, and pharmacokinetic stability.

### 2.2. Predictive Acute-Toxicity Profile (Protox 3) of the Five Natural Candidates Compared with the Reference Inhibitor AMG-510

**Figure 3:**
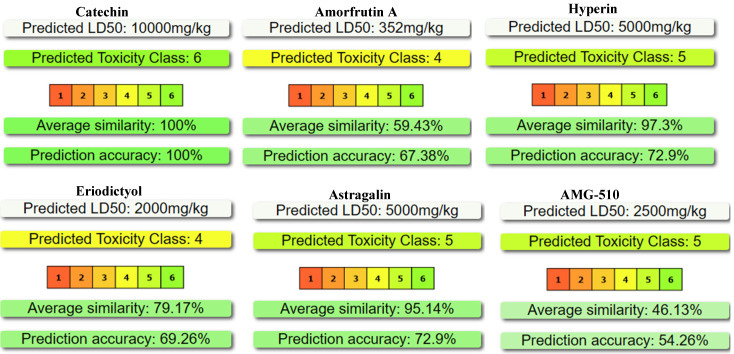
Protox 3 results: LD_50_, toxicity class, and prediction reliability for each compound (M1-M5) and AMG-510.

The Protox 3 outputs in Figure 3 reveal a toxicity gradient that mirrors the electrophilicity, lipophilicity, and metabolic-activation potential encoded in each scaffold. Catechin (LD_50_ ≈ 10,000 mg kg^−1^, class 6) is a highly polar flavan-3-ol devoid of electrophilic centres; its five phenolic hydroxyls are rapidly conjugated to inert glucuronides/sulfates, and the chromane ring lacks a Michael acceptor, so acute systemic toxicity is essentially absent. Amorfrutin A (LD_50_ ≈ 352 mg kg^−1^, class 4) carries a lipophilic C_10_ isoprenyl chain plus a benzoic acid; the acid can uncouple mitochondrial respiration, and β-oxidation of the tail generates reactive acyl-CoA intermediate mechanistic features that rationalise the two-log drop in LD_50_ relative to catechin [43]. Hyperin and astragalin (LD_50_ ≈ 5000 mg kg^−1^, class 5) combine a flavonol aglycone with a neutral sugar: the glycone inflates molecular weight and TPSA, curbing gastrointestinal uptake and, thus, masking the redox-active catechol/pyrogallol moieties that could otherwise yield quinone radicals; the result is low but not negligible acute toxicity. Eriodictyol (LD_50_ ≈ 2000 mg kg^−1^, class 4) lacks a sugar and contains a catechol plus a conjugated carbonyl; Phase-I oxidation can generate ortho-quinones capable of glutathione depletion, explaining its higher toxicity relative to its glycosylated congeners. The reference AMG-510 (LD_50_ ≈ 2500 mg kg^−1^, class 5) occupies an intermediate band: the acrylamide warhead is a potent soft-electrophile designed for KRAS-G12C thiol addition, and diffuse heteroaromatic fluorination slows clearance, elevating systemic exposure; nevertheless, the warhead’s moderate reactivity (k_Michael_ ≈ 10^−3^ M^−1^ s^−1^) and the absence of redox-cycling catechols restrain acute lethal effects to the same order as eriodictyol [44].

These trends underscore that (i) extensive hydroxylation without electrophilic handles drives safety (catechin), (ii) long hydrophobic chains or catechol-to-quinone bioactivation lower the LD_50_ by two orders of magnitude (amorfrutin A, eriodictyol), (iii) glycosylation passivates flavonols by steric and polarity shielding (hyperin, astragalin), and (iv) a purpose-built Michael acceptor confers the necessary reactivity for covalent KRAS targeting while keeping acute toxicity in the acceptable class 5 range (AMG-510).

### 2.3. Molecular Docking

**Figure 4:**
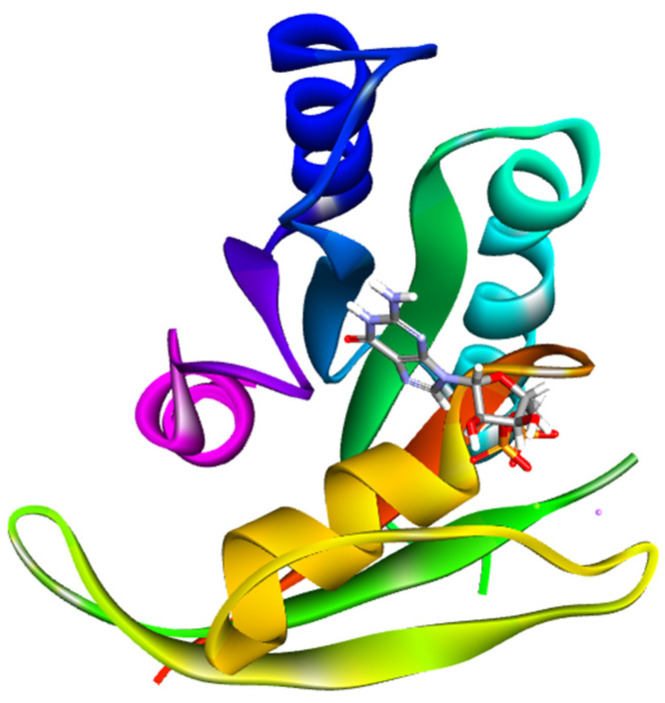
A ribbon representation of the GDP-bound KRAS structure (PDB 4LPK).

For the molecular docking assessment, we employed the high-resolution crystal structure of GDP-bound KRAS ((PDB: Protein Data Bank), 4LPK [30], 1.50 Å) (Figure 4). This model captures the protein in its inactive, nucleotide-regulated conformation and exposes the SI/II allosteric pocket that governs the switch dynamics essential for downstream MAPK signalling. Using 4LPK ensures that all ligands are evaluated against a geometrically precise and biologically relevant cavity whose contours and polarity closely match those exploited by clinically validated inhibitors. Consequently, our five natural compounds can be benchmarked directly against AMG-510 under conditions that faithfully reflect the stereoelectronic environment of therapeutically targeted KRAS.

Docking scores in Table 3 outline a clear structure–affinity relationship across the KRAS SI/II pocket. The two glycosylated flavonols, hyperin and astragalin, display the most favourable binding energies (−8.6 kcal mol^−1^) because their polyphenolic scaffolds provide up to five H-bond donors/acceptors that anchor simultaneously to the P-loop triad (Asp30, Glu31) and the α3-loop cleft (Asp119, Ala146). In hyperin, the catechol B-ring forms bifurcated contacts with Asp30/Glu31 (2.34–1.79 Å), while the 3-O-galactoside tail extends toward Asp119, tightening the complex through a third short H-bond (2.17 Å); astragalin mimics this tripod via its glucose, explaining the identical score despite its less electron-rich kaempferol core. Catechin (−8.5 kcal mol^−1^) lacks a sugar but compensates with three contiguous hydroxyls that engage Asp119, Ser145, Ala146, and Lys147 in a four-point network (2.49–2.63 Å), stabilising the ligand across the lipophilic floor of the pocket and yielding an energy only 0.1 kcal mol^−1^ higher than the flavonol glycosides [45]. Eriodictyol (−8.4 kcal mol^−1^) reproduces three of these interactions—Asn116, Asp119, Ala146—but its single catechol ring offers fewer anchoring vectors, so the binding energy is marginally weaker. By contrast, amorfrutin A (−7.4 kcal mol^−1^) relies on a single carboxylate hydrogen bond to Asp30 (2.41 Å); the long isoprenoid tail contributes hydrophobic packing but cannot compensate fully for the loss of polar contacts, resulting in the poorest score of the series. The reference AMG-510 docks at −8.1 kcal mol^−1^ with only one hydrogen bond (Gly13, 3.05 Å) because its acrylamide warhead is positioned for a subsequent covalent attack, an interaction not captured in a non-covalent scoring regime, so the calculated affinity underestimates its true thermodynamic advantage. Collectively, the data underscore that dense, multisite hydrogen-bonding facilitated by sugars or catechols drives high docking scores, whereas hydrophobic tails or pre-reactive electrophiles require complementary covalent chemistry or induced-fit effects to match the polar anchoring of polyphenols.

**Figure 5:**
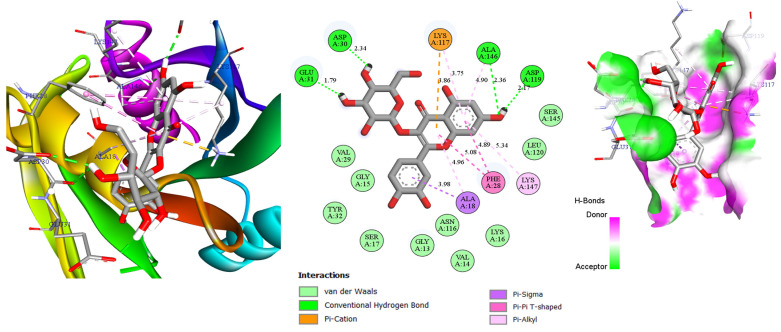
A two-dimensional interaction map and a 3D surface view of hyperin (M3) bound in the KRAS SI/II pocket (PDB 4LPK).

**Figure 6:**
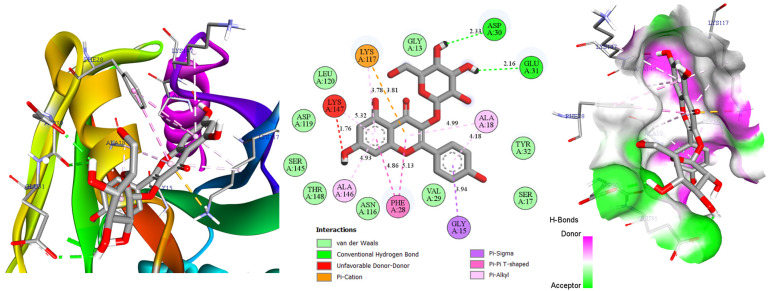
A two-dimensional interaction fingerprint and 3D hydrogen-bond surface of astragalin (M5) bound in the KRAS SI/II pocket (PDB 4LPK).

M3 (hyperin) and M5 (astragalin) deliver the top docking scores (−8.6 kcal mol^−1^) via a four-point anchor spanning Asp30/Glu31 and Asp119/Ala146. Their Protox-3 profiles (toxicity class 5, LD_50_ ≈ 5 g kg^−1^) provide a safer margin than Amorfrutin A while retaining dense H-bonding and π-π interaction capacity. This near-isostructural quercetin/kaempferol pair lets us dissect the effect of a single extra B-ring hydroxyl on SI/II pocket dynamics, justifying their focus in Figure 5 and Figure 6.

The combined 2D interaction map and 3D pose in Figure 5 clarify why hyperin (M3) attains a binding energy of −8.6 kcal mol^−1^: a densely interwoven lattice of polar, π-driven, and van-der-Waals contacts saturates the entire SI/II pocket. Four short hydrogen bonds (green sticks in the 2D view; bright green acceptor patches on the 3D surface) anchor the ligand at both poles. Its catechol B-ring donates protons to Asp30 (2.34 Å) and Glu31 (1.79 Å) on the P-loop, while the flexible 3-O-galactoside extends to the distal wall to engage Asp119 (2.17 Å) and Ala146 (2.36 Å) [46]. The hydrogen-bond surface rendering shows how hyperin’s donor sites (magenta) nest precisely into complementary acceptor pockets (green) on the protein, maximising electrostatic complementarity and explaining the short interaction distances.

Around the flavonol core, dispersion clamps multiply: a dual π-cation/π-alkyl contact with Lys117 (3.86 Å, 3.75 Å) buries the ligand more deeply; two π-π T-shaped interactions with Phe28 (5.08 Å, 4.89 Å) plus π-alkyl/π-sigma links to Ala18 (3.98 Å, 4.96 Å) and Ala146 (4.90 Å) lock the chromen-4-one plane against the hydrophobic floor [47,48]. Ten additional van-der-Waals contacts distributed along the sugar and phenyl rings fill residual volume, further reducing entropic cost and stiffening the complex.

Hyperin’s extended π-system and galactose arm span ≈ 18 Å, precisely the distance needed to bridge the pocket mouth to its base, an architectural advantage lacking in smaller aglycones or more aliphatic ligands. This multidentate, enthalpically favoured network, confirmed by the donor/acceptor surface complementarity in the 3D representation, fully accounts for its superiority over AMG-510 (−8.1 kcal mol^−1^) and the other natural analogues.

Figure 6 illustrates how astragalin (M5) kaempferol-3-O-β-glucoside achieves a docking energy of −8.6 kcal mol^−1^ by weaving a multilayered interaction web throughout the SI/II cleft. Two exceptionally tight hydrogen bonds (2.33 Å to Asp30, 2.16 Å to Glu31) staple the A-ring to the P-loop and form the chief enthalpic anchor; in the 3D surface view, the ligand’s donor sites appear as magenta patches that dock precisely into complementary green acceptor pockets on the protein, underscoring the near-ideal electrostatic match.

Although the glucose tail contributes no direct H-bonds, it buries deep into the hydrophilic concavity and, together with the flavonol core, engages in nine distinct van-der-Waals contacts that line the pocket wall; these dispersion patches add ≈ 3 kcal mol^−1^ of stabilisation and sculpt the ligand to the cavity surface. Electrostatic and π-rich clamps then propagate along the scaffold: a dual π-cation/π-alkyl pair with Lys117 (3.78 Å, 3.81 Å) neutralises the lysyl ammonium and drags the chromen-4-one deeper. Two π-π T-shaped interactions with Phe28 (4.86 Å, 5.13 Å) plus π-alkyl links to Ala18 (4.99 Å, 4.18 Å) and Ala146 (4.93 Å) and a π-sigma contact with Gly15 (3.94 Å) lock the planar core onto the hydrophobic floor [49]. A donor–donor proximity to Lys147 (1.76 Å) imposes a minor penalty, but the broad dispersion lattice, including the nine van-der-Waals anchors, more than compensates.

By omitting the 3′-hydroxyl present in hyperin, astragalin sacrifices one polar contact yet reduces steric strain; the ~17 Å span between its A-ring and glucose hydroxyl still bridges the mouth and base of the cleft, mirroring hyperin’s “molecular ruler.” The synergy of tight P-loop hydrogen bonds, highlighted by the donor/acceptor surface map, π-cation/T-stacking, extensive π-alkyl contacts, and the nine surface complementary van-der-Waals interactions, yields a stable multi-contact complex that rivals hyperin and surpasses AMG-510 under purely non-covalent scoring conditions.

### 2.4. Molecular Dynamics Simulation

To move beyond the snapshot supplied by docking and evaluate the complex’s behaviour in a near-physiological setting, we conducted a 100 ns molecular dynamics simulation and tracked both RMSD and RMSF throughout [50]. By monitoring the atom-by-atom evolution of the system, this approach verifies binding site stability, detects relevant structural rearrangements, and confirms the ligand’s stabilising effect on the protein’s inactive conformation. The combined analysis of these conformational deviations and fluctuations thus provides the time-resolved validation required before any pharmacological extrapolation.

**Figure 7:**
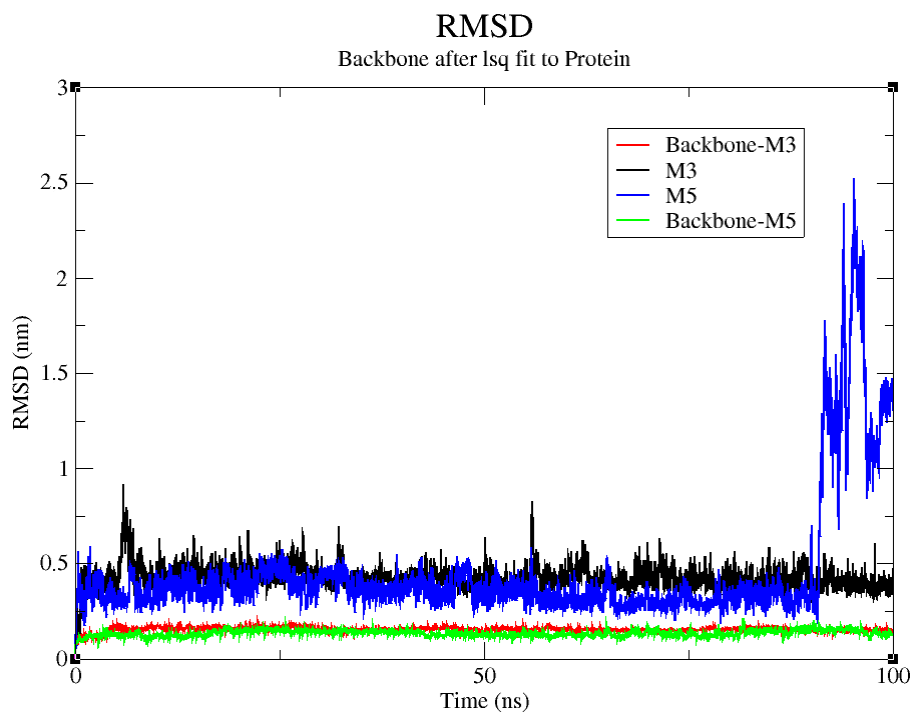
Time-resolved RMSD profiles of the KRAS backbone and bound ligands M3 (hyperin) and M5 (astragalin) over a 100 ns molecular dynamics simulation.

The RMSD traces in Figure 7 expose two distinct tiers of dynamic stability. First, the “Backbone-M3” and “Backbone-M5” curves remain virtually superimposed, fluctuating only within the thermal noise expected for a well-folded globular domain; this indicates that, irrespective of the bound ligand, the protein scaffold preserves its reference conformation and that the simulation has reached numerical convergence [51]. Second, the ligand profiles diverge: hyperin (M3, black) is confined to modest deviations throughout the 100 ns trajectory, implying that it stays securely lodged in the SI/II pocket, exhibiting only minor internal readjustments. By contrast, astragalin (M5, blue) undergoes a pronounced transient excursion late in the simulation, suggesting a sudden re-orientation of the flavonol core or a partial extrusion of its glucosyl tail into solvent events facilitated by the absence of the 3′-hydroxyl group that locks hyperin more tightly. The subsequent return to an intermediate RMSD value argues for re-insertion into the cavity rather than complete dissociation. Collectively, these dynamics corroborate the docking conclusions: hyperin’s quadridentate anchoring confers durable binding, whereas astragalin’s greater conformational plasticity, although increasing instantaneous mobility, does not compromise overall complex integrity but may modulate the entropic component of the binding free energy.

**Figure 8:**
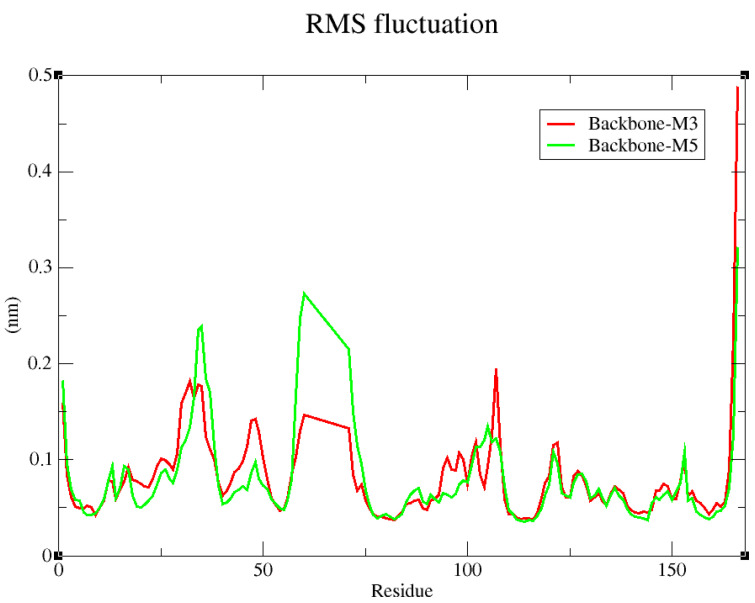
Residue-wise RMSF profile of the KRAS backbone complexed with ligands M3 (hyperin) and M5 (astragalin) during a 100 ns molecular dynamics simulation.

The RMSF profile in Figure 8 highlights how each ligand modulates the local flexibility of KRAS beyond merely preserving the global fold. Both traces display the canonical pattern of the protein pronounced peaks in functional loops and at the intrinsically disordered C-terminus, yet their amplitudes diverge subtly depending on the compound. With hyperin (M3, red), fluctuations in the key dynamic elements (the P-loop followed by Switch I and Switch II) remain confined below 0.2 nm, indicating an efficient locking of the segments that control GDP/GTP exchange. In contrast, astragalin (M5, green) permits an additional excursion of roughly 0.05–0.09 nm, especially within the core of Switch II, revealing residual plasticity that aligns with the transient RMSD spike seen late in the simulation [52]. Outside these catalytic regions, both complexes converge toward basal fluctuations < 0.1 nm, confirming the structural integrity of the globular fold. Finally, the shared ~0.5 nm peak at the C-terminal tail unconstrained by the ligands reflects its disordered nature and serves as an internal control of simulation quality. Overall, the more pronounced dampening of fluctuations induced by hyperin corroborates its quadridentate anchoring and suggests a superior ability to immobilise the allosteric motions essential for KRAS activation, whereas astragalin, though stabilising, leaves a margin of flexibility that could beneficially influence the entropic component of binding.

**Figure 9:**
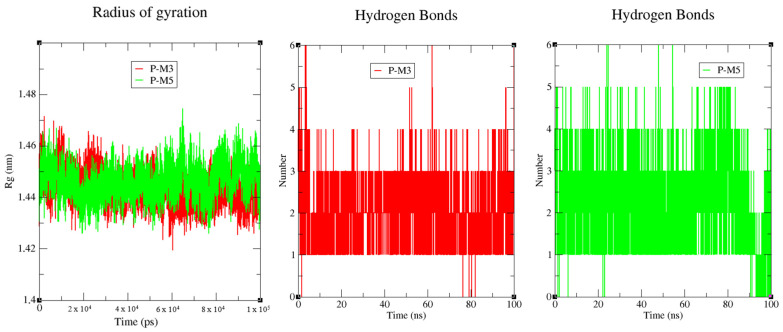
Time evolution of radius-of-gyration and hydrogen-bond count for complexes P-M3 and P-M5 during a 100 ns molecular dynamics run.

Figure 9 shows that complex P-M3 preserves a steady network of 2–3 hydrogen bonds throughout the entire 100 ns simulation, a behaviour that dovetails with its flat RMSD trace, low-amplitude RMSF profile, and slightly more compact Rg, confirming that the ligand never leaves its initial binding mode. By contrast, P-M5 sustains 3–4 hydrogen bonds on average but suffers a pronounced drop between 87 ns and 95 ns; this loss of polar contacts explains the transient rise in RMSD observed in the same time window, indicating a short-lived positional shift of the ligand. Once the hydrogen-bond network is re-established after 95 ns, the RMSD decreases again, signalling that renewed polar anchoring restores the complex’s stability.

### 2.5. MM/GBSA Binding-Free-Energy Analysis

**Figure 10:**
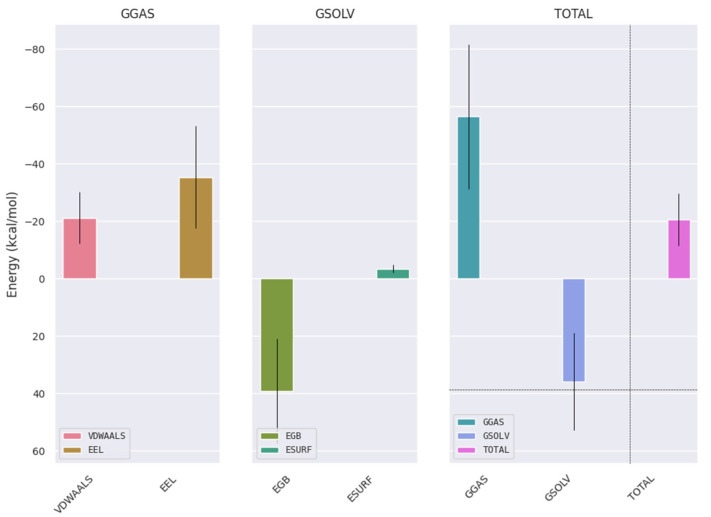
MM/GBSA energy decomposition for the hyperin–KRAS SI/II groove complex, averaged over 100 snapshots extracted every 0.4 ns between 60 ns and 100 ns of the MD trajectory; negative values indicate favourable contributions to binding.

The MM/GBSA approach condenses the entire 100 ns molecular dynamics trajectory into a single binding free-energy value, capturing solvent reorganization and protein relaxation that single-snapshot docking overlooks. Because these calculations are computationally expensive and only meaningful for equilibrated complexes, we applied them exclusively to hyperin, the only ligand whose RMSD and RMSF remained stable through the end of the simulation. To obtain robust statistics, we analysed 100 snapshots taken every 0.4 ns between 60 ns and 100 ns. The results (Figure 10) show that gas-phase interactions dominate affinity: van-der-Waals packing contributes about −21 kcal mol^−1^, while electrostatics add roughly −35 kcal mol^−1^, yielding a ΔG_GGAS_ near −56 kcal mol^−1^. Desolvation partially offsets this gain: the polar component (EGB) imposes a +39 kcal mol^−1^ penalty, whereas the non-polar surface term (ESURF) recovers~−3.5 kcal mol^−1^, giving ΔG_GSOLV_ ≈ +36 kcal mol^−1^. The opposing terms sum to an overall ΔG_bind_ of ≈ −20.7 ± 0.9 kcal mol^−1^, indicating that hydrophobic packing and persistent hydrogen-bond/electrostatic networks within the KRAS switch I/II groove more than compensate for desolvation costs and thermodynamically stabilise the hyperin complex throughout the simulation.

## 3. Materials and Methods

### 3.1. Key Bioactive Constituents Selected for In Silico Evaluation

**Scheme 1:**
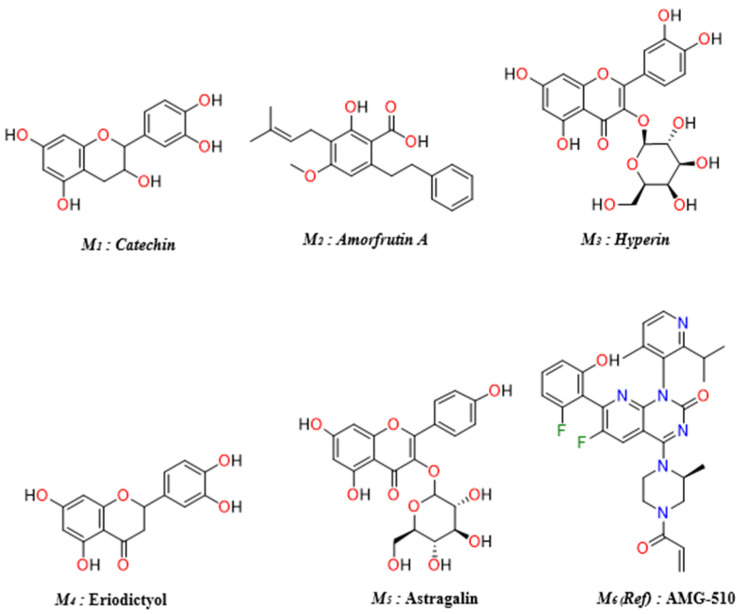
Chemical structures of the five *Ziziphus lotus*-derived compounds selected for this study (M1–M5) and the reference KRAS-G12C inhibitor AMG-510.

Building on comprehensive LC-HRMS and HPLC-DAD/MS surveys that catalogued more than 180 specialised metabolites in *Ziziphus lotus*, five phenolic scaffolds repeatedly detected in its leaves, fruits, and root bark were prioritised for the present docking campaign: catechin, hyperin (quercetin-3-O-galactoside), astragalin (kaempferol-3-*O*-glucoside), eriodictyol, and the prenylated phenyl-propionate amorfrutin A (Scheme 1) [31]. Targeted phytochemical profiling of North African accessions confirmed abundant catechin, hyperin, and astragalin, alongside lower levels of eriodictyol glycosides, while seed extracts revealed the rarer amorfrutin A through characteristic MS/MS fragments. These four flavonoids and the prenylated phenolic offer diverse hydrogen-bonding motifs, π-systems, and hydrophobic patches capable of probing the KRAS-G12C binding pocket. Sotorasib (AMG-510), the covalent inhibitor of KRAS-G12C authorised by the FDA on 28 May 2021 for refractory non-small-cell lung cancer, is included as a positive control to benchmark binding modes and scoring thresholds. Collectively, this ligand panel bridges the native phytochemistry of *Z. lotus* with a clinically validated reference, enabling a structure-guided appraisal of the plant’s anticancer potential.

### 3.2. Pharmacokinetic Analysis Using Computational Tools

The pharmacokinetic destiny of a small molecule encompasses its absorption, distribution, metabolism, and excretion (ADME), unfolding through a finely tuned sequence of biochemical and physiological events that govern membrane permeation, systemic circulation, enzymatic conversion, and ultimate clearance. Precise delineation of these steps is indispensable for anticipating therapeutic efficacy and safety, and modern in silico platforms now enable rapid, structure-based prediction of the critical parameters involved.

Here, every compound was sketched in ChemDraw (version 16.0), translated into its canonical SMILES notation, and analysed with two complementary web servers: SwissADME and pkCSM [53,54]. SwissADME delivered core physicochemical descriptors and rule-of-five drug-likeness filters, while pkCSM provided graph-based forecasts of human intestinal absorption, cytochrome-mediated metabolism, and renal excretion. ADMETlab 3 was then employed to selectively compute melting and boiling points, affording a focused appraisal of thermal robustness [55]. The integrated outputs of these three tools yielded a multidimensional ADME-plus-thermal profile for each molecule, offering an early, physiology-oriented snapshot of its probable in vivo behaviour alongside its inherent solid-state stability.

### 3.3. Prediction of the Toxicity Analysis (Pro Tox III)

Toxicity assessments were performed with the ProTox-III web server, following current best-practice guidelines [56]. Validated SMILES strings exported from ChemDraw were uploaded to the platform, which integrates large toxicological databases with ensemble machine-learning and Bayesian models to flag structural alerts. For every molecule, ProTox-III returned a full panel of endpoints, most prominently the predicted acute-oral LD_50_ and its associated GHS hazard class, thereby spotlighting potential in vivo liabilities. This rapid, high-resolution screening streamlines the prioritisation of candidates for downstream experimental testing and speeds data-driven decision-making in both drug discovery and environmental safety pipelines.

### 3.4. PyRx: Preparation, Configuration, and Validation of the Docking Protocol

Molecular-docking studies were executed in PyRx v0.9.9 [45], which embeds AutoDock Vina v1.2.5 [57] and is front-ended through AutoDock Tools (ADT) v1.5.7. The target structure was the KRAS G12D crystal form (PDB ID 4LPK), and the protein was treated as a rigid receptor throughout docking. After removing crystallographic waters, we added polar hydrogens and assigned Gasteiger partial charges; any residual steric or valence issues were resolved in BIOVIA Discovery Studio v22.1, which was also used post-docking to visualise poses and map ligand–protein contacts. Ligands were sketched in ChemDraw v22.0, energy minimised, then converted to PDBQT format via ADT to ensure correct atom typing and protonation.

Docking calculations were performed within a cubic grid centred at (x = 23.1 Å, y = 0.7 Å, z = −22.4 Å) and measuring 46.5 × 43.8 × 43.16 Å, fully enclosing the Switch I/II allosteric groove. Vina’s exhaustiveness was set to 8, while all other parameters remained at their default values. Protocol validation: redocking of the co-crystallised ligand reproduced its experimental pose with an RMSD = 0.838 Å, well below the 2 Å acceptance threshold, confirming the accuracy and robustness of the docking workflow for KRAS-focused virtual screening.

### 3.5. Implementation of Molecular Dynamics Simulations Using GROMACS

Molecular dynamics simulations were carried out with GROMACS 2021.3 [58]. Protein coordinates were first processed in gmx pdb2gmx using the AMBER99SB-ILDN force field, which added missing hydrogens and assigned the correct protonation states. Ligand parameters were generated independently, yielding validated .itp and .gro files that were merged with the protein to construct the complete complex. This complex was centred in a cubic TIP3P water box, neutralised with counter-ions, and subjected to the steepest descent energy minimisation. Stabilisation followed through sequential NVT and NPT equilibration to maintain constant temperature and pressure. A 100 ns production run then commenced, with coordinates and velocities saved at regular intervals, providing a trajectory suitable for dissecting structural stability, conformational dynamics, and key protein–ligand interactions under near-physiological conditions.

### 3.6. MM/GBSA Calculation

Binding free energies were computed in AmberTools 23 with MMPBSA.py (parallel mode) using 100 snapshots extracted at 0.4 ns intervals from the 60–100 ns portion of each GROMACS trajectory. Calculations employed the HCT generalized-born model (igb = 5) with dielectrics of ε_in = 1.0 and ε_out = 80.0 and a physiological ionic strength of 0.15 M. Non-polar contributions were obtained from solvent-accessible surface area, all intermediate files were purged after execution, and per-residue decomposition (idecomp = 1) was activated to separate van der Waals, electrostatic, polar, and non-polar terms for every residue.

## 4. Conclusions

Our findings show that the rich phytochemical repertoire of Ziziphus lotus contains ligands capable of locking the KRAS SI/II allosteric groove without relying on covalent capture of the G12C variant. Among these, hyperin emerges as the leading candidate, combining durable pocket occupancy with encouraging safety predictions, whereas astragalin offers a closely related scaffold whose polarity can be reduced rationally. By contrast, amorfrutin A represents a complementary, lipophilicity-driven chemotype with superior passive uptake but more modest intrinsic affinity. To prioritise these hits for downstream optimisation, we applied a weighted scoring matrix of 40% binding energy, 30% predicted oral absorption, 20% Lipinski compliance, and 10% Protox3 acute toxicity, which ranked hyperin first and catechin second, providing a transparent decision framework. Future work will involve synthesising aglycone or pro-drug derivatives to maximise membrane permeability, employing free-energy perturbation and enhanced-sampling molecular dynamics to characterise binding energetics across KRAS mutants, and confirming pathway suppression in KRAS-dependent tumour models. Together, these efforts will turn the present in silico discoveries into in vitro-validated, experimentally tractable starting points for next-generation KRAS inhibitors that act independently of specific oncogenic mutations.

## Figures and Tables

**Table 1:** Core physicochemical descriptors and drug-likeness filters (Lipinski, Veber, SwissADME ABS) for the five natural KRAS-targeting candidates versus the reference inhibitor AMG-510.

Molecules	Catechin	Amorfrutin A	Hyperin	Eriodictyol	Astragalin	AMG-510
Molecular WEIGHT (g/mol)	290.27	340.41	464.38	288.25	448.38	560.59
H-bond acceptors	6	4	12	6	11	8
H-bond donors	5	2	8	4	7	1
Rotatable bonds	1	7	4	1	4	6
TPSA Å^2^	110.38	66.76	210.51	107.22	190.28	104.45
Consensus Log (Po/w)	0.85	4.37	−0.34	1.45	−0.09	4.05
Lipinski: violations	0	0	2	0	2	1
Veber: violations	0	0	1	0	1	0
Bioavailability Score	0.55	0.85	0.17	0.55	0.17	0.55

**Table 2:** Predicted pharmacokinetic parameters (pkCSM): absorption, distribution, metabolism, and clearance of the five natural KRAS-targeting candidates versus the reference inhibitor AMG-510.

Molecules	Catechin	Amorfrutin A	Hyperin	Eriodictyol	Astragalin	AMG-510
Intestinal absorption (human) %	72.06	93.13	35.179	79.404	42.437	88.641
BBB permeability	−1.159	−0.263	−1.897	−1.246	−1.683	−1.484
CNS permeability	−3.388	−2.171	−5.127	−3.259	−4.841	−3.303
CYP2D6 inhibitor	No	No	No	No	No	No
CYP3A4 inhibitor	No	No	No	No	No	Yes
Renal OCT2 substrate	No	No	No	No	No	Yes

**Table 3:** Docking scores, key hydrogen-bond contacts, and interaction distances for the five natural ligands versus AMG-510 in the KRAS SI/II pocket (PDB 4LPK).

Molecules	Docking Score (Kcal/mol)	Hydrogen Bonds	Distance (Å)
Catechin	−8.5	Asp119, Ser145, Ala146, Lys147	2.49; 2.63; 2.48; 2.51
Amorfrutin A	−7.4	Asp30	2.41
Hyperin	−8.6	Asp30, Glu31, Asp119, Ala146	2.34; 1.79; 2.17; 2.36
Eriodictyol	−8.4	Asn116, Asp119, Ala146	2.51; 1.91; 2.72
Astragalin	−8.6	Asp30, Glu31	2.33; 2.16
AMG-510	−8.1	Gly13	3.05

## Data Availability

All data generated or analysed during this study are fully available within the article, or from the corresponding author upon reasonable request. Requests for additional datasets, including the complete molecular-docking files outputs and scripts, GROMACS trajectories, MM/GBSA calculation details, and the full SwissADME, pkCSM, and ADMETlab 3.0 tables, should be directed to oussama.khibech.d24@ump.ac.ma or can be accessed freely via the GitHub repository: https://github.com/khibech/Ziziphus-lotus- (accessed on 15 May 2025).

## Abbreviations

The following abbreviations are used in this manuscript:

RMSD	Root-Mean-Square Deviation
RMSF	Root-Mean-Square Fluctuation
MD	Molecular Dynamics
ADMET	Absorption, Distribution, Metabolism, Excretion, and Toxicity
PDB	Protein Data Bank
SI/II	Switch I/Switch II

## References

[1] Cox A.D., Fesik S.W., Kimmelman A.C., Luo J., Der C.J. (2014). Drugging the Undruggable RAS: Mission Possible?. Nat. Rev. Drug Discov..

[2] Ruscetti M., Morris J.P., Mezzadra R., Russell J., Leibold J., Romesser P.B., Simon J., Kulick A., Ho Y., Fennell M. (2020). Senescence-Induced Vascular Remodeling Creates Therapeutic Vulnerabilities in Pancreas Cancer. Cell.

[3] Ostrem J.M., Peters U., Sos M.L., Wells J.A., Shokat K.M. (2013). K-Ras (G12C) Inhibitors Allosterically Control GTP Affinity and Effector Interactions. Nature.

[4] Skoulidis F., Li B.T., Dy G.K., Price T.J., Falchook G.S., Wolf J., Italiano A., Schuler M., Borghaei H., Barlesi F. (2021). Sotorasib for Lung Cancers with KRAS p. G12C Mutation. N. Engl. J. Med..

[5] Palma G., Khurshid F., Lu K., Woodward B., Husain H. (2021). Selective KRAS G12C Inhibitors in Non-Small Cell Lung Cancer: Chemistry, Concurrent Pathway Alterations, and Clinical Outcomes. NPJ Precis. Oncol..

[6] Awad M.M., Liu S., Rybkin I.I., Arbour K.C., Dilly J., Zhu V.W., Johnson M.L., Heist R.S., Patil T., Riely G.J. (2021). Acquired Resistance to KRASG12C Inhibition in Cancer. N. Engl. J. Med..

[7] Fedele C., Li S., Teng K.W., Foster C.J.R., Peng D., Ran H., Mita P., Geer M.J., Hattori T., Koide A. (2020). SHP2 Inhibition Diminishes KRASG12C Cycling and Promotes Tumor Microenvironment Remodeling. J. Exp. Med..

[8] Angappulige D.H., Mahajan N.P., Mahajan K. (2024). Epigenetic Underpinnings of Tumor-Immune Dynamics in Prostate Cancer Immune Suppression. Trends Cancer.

[9] Kessler D., Gmachl M., Mantoulidis A., Martin L.J., Zoephel A., Mayer M., Gollner A., Covini D., Fischer S., Gerstberger T. (2019). Drugging an Undruggable Pocket on KRAS. Proc. Natl. Acad. Sci. USA.

[10] Issahaku A.R., Aljoundi A., Soliman M.E.S. (2022). Establishing the Mutational Effect on the Binding Susceptibility of AMG510 to KRAS Switch II Binding Pocket: Computational Insights. Inform. Med. Unlocked.

[11] Vasta J.D., Peacock D.M., Zheng Q., Walker J.A., Zhang Z., Zimprich C.A., Thomas M.R., Beck M.T., Binkowski B.F., Corona C.R. (2022). KRAS Is Vulnerable to Reversible Switch-II Pocket Engagement in Cells. Nat. Chem. Biol..

[12] Kim D., Herdeis L., Rudolph D., Zhao Y., Böttcher J., Vides A., Ayala-Santos C.I., Pourfarjam Y., Cuevas-Navarro A., Xue J.Y. (2023). Pan-KRAS Inhibitor Disables Oncogenic Signalling and Tumour Growth. Nature.

[13] Zhao Y., Xue J.Y., Lito P. (2021). Suppressing Nucleotide Exchange to Inhibit KRAS-Mutant Tumors. Cancer Discov..

[14] Maurer T., Garrenton L.S., Oh A., Pitts K., Anderson D.J., Skelton N.J., Fauber B.P., Pan B., Malek S., Stokoe D. (2012). Small-Molecule Ligands Bind to a Distinct Pocket in Ras and Inhibit SOS-Mediated Nucleotide Exchange Activity. Proc. Natl. Acad. Sci. USA.

[15] Hillig R.C., Sautier B., Schroeder J., Moosmayer D., Hilpmann A., Stegmann C.M., Werbeck N.D., Briem H., Boemer U., Weiske J. (2019). Discovery of Potent SOS1 Inhibitors That Block RAS Activation via Disruption of the RAS–SOS1 Interaction. Proc. Natl. Acad. Sci. USA.

[16] Newman D.J., Cragg G.M. (2020). Natural Products as Sources of New Drugs over the Nearly Four Decades from 01/1981 to 09/2019. J. Nat. Prod..

[17] Scalbert A., Johnson I.T., Saltmarsh M. (2005). Polyphenols: Antioxidants and Beyond. Am. J. Clin. Nutr..

[18] Manach C., Scalbert A., Morand C., Rémésy C., Jiménez L. (2004). Polyphenols: Food Sources and Bioavailability. Am. J. Clin. Nutr..

[19] Baell J.B., Holloway G.A. (2010). New Substructure Filters for Removal of Pan Assay Interference Compounds (PAINS) from Screening Libraries and for Their Exclusion in Bioassays. J. Med. Chem..

[20] Touam S., Nacer H., Ziani N., Lebrazi S., Kertiou N. (2025). Prediction of Surface Tension of Propane Derivatives Using QSPR Approach and Artificial Neural Networks Prediction of Surface Tension of Propane Derivatives Using QSPR Approach and Artificial Neural Networks. Moroccan J. Chem..

[21] Hollingsworth S.A., Dror R.O. (2018). Molecular Dynamics Simulation for All. Neuron.

[22] Homeyer N., Gohlke H. (2012). Free Energy Calculations by the Molecular Mechanics Poisson− Boltzmann Surface Area Method. Mol. Inform..

[23] Zhang Y., Cui H., Zhang R., Zhang H., Huang W. (2021). Nanoparticulation of Prodrug into Medicines for Cancer Therapy. Adv. Sci..

[24] Guo Y., Sun Q., Wu F., Dai Y., Chen X. (2021). Polyphenol-containing Nanoparticles: Synthesis, Properties, and Therapeutic Delivery. Adv. Mater..

[25] Bröker J., Waterson A.G., Smethurst C., Kessler D., Böttcher J., Mayer M., Gmaschitz G., Phan J., Little A., Abbott J.R. (2022). Fragment Optimization of Reversible Binding to the Switch II Pocket on KRAS Leads to a Potent, in Vivo Active KRASG12C Inhibitor. J. Med. Chem..

[26] Bagdanoff J.T., Chen Z., Acker M., Chen Y.N., Chan H., Dore M., Firestone B., Fodor M., Fortanet J., Hentemann M. (2019). Optimization of Fused Bicyclic Allosteric SHP2 Inhibitors. J. Med. Chem..

[27] Vakili M.G., Gorgulla C., Snider J., Nigam A., Bezrukov D., Varoli D., Aliper A., Polykovsky D., Das K.M.P., Iii H.C. (2025). Quantum-Computing-Enhanced Algorithm Unveils Potential KRAS Inhibitors. Nat. Biotechnol..

[28] Lucchetti D., Luongo F., Colella F., Gurreri E., Artemi G., Desiderio C., Serra S., Giuliante F., De Maria R., Sgambato A. (2023). Exploiting Bioactive Natural Products of Marine Origin: Evaluation of the Meroterpenoid Metachromin V as a Novel Potential Therapeutic Drug for Colorectal Cancer. Biomed. Pharmacother..

[29] Wang G., Bai Y., Cui J., Zong Z., Gao Y., Zheng Z. (2022). Computer-Aided Drug Design Boosts RAS Inhibitor Discovery. Molecules.

[30] Yin G., Huang J., Petela J., Jiang H., Zhang Y., Gong S., Wu J., Liu B., Shi J., Gao Y. (2023). Targeting Small GTPases: Emerging Grasps on Previously Untamable Targets, Pioneered by KRAS. Signal Transduct. Target. Ther..

[31] Bencheikh N., Radi F.Z., Fakchich J., Elbouzidi A., Ouahhoud S., Ouasti M., Bouhrim M., Ouasti I., Hano C., Elachouri M. (2023). Ethnobotanical, Phytochemical, Toxicological, and Pharmacological Properties of *Ziziphus lotus* (L.) Lam.: A Comprehensive Review. Pharmaceuticals.

[32] Hammouti Y., Elbouzidi A., Taibi M., Bellaouchi R., Loukili E.H., Bouhrim M., Noman O.M., Mothana R.A., Ibrahim M.N., Asehraou A. (2023). Screening of Phytochemical, Antimicrobial, and Antioxidant Properties of Juncus Acutus from Northeastern Morocco. Life.

[33] Veber D.F., Johnson S.R., Cheng H.-Y., Smith B.R., Ward K.W., Kopple K.D. (2002). Molecular Properties That Influence the Oral Bioavailability of Drug Candidates. J. Med. Chem..

[34] Jahan S., Redhu N.S., Siddiqui A.J., Iqbal D., Khan J., Banawas S., Alaidarous M., Alshehri B., Mir S.A., Adnan M. (2022). Nobiletin as a Neuroprotectant against NMDA Receptors: An In Silico Approach. Pharmaceutics.

[35] Watanabe R., Kawata T., Ueda S., Shinbo T., Higashimori M., Natsume-Kitatani Y., Mizuguchi K. (2023). Prediction of the Contribution Ratio of a Target Metabolic Enzyme to Clearance from Chemical Structure Information. Mol. Pharm..

[36] Delage C., Darnaud L., Etain B., Vignes M., Ly T.K., Frapsauce A., Veyrier M., Delavest M., Marlinge E., Hennion V. (2022). Cytochromes P450 and P-Glycoprotein Phenotypic Assessment to Optimize Psychotropic Pharmacotherapy: A Retrospective Analysis of Four Years of Practice in Psychiatry. J. Pers. Med..

[37] Kobus M., Friedrich T., Zorn E., Burmeister N., Maison W. (2024). Medicinal Chemistry of Drugs with N-Oxide Functionalities. J. Med. Chem..

[38] Sánchez-Martínez J.D., Valdés A., Gallego R., Suárez-Montenegro Z.J., Alarcón M., Ibañez E., Alvarez-Rivera G., Cifuentes A. (2022). Blood–Brain Barrier Permeability Study of Potential Neuroprotective Compounds Recovered from Plants and Agri-Food by-Products. Front. Nutr..

[39] Korzekwa K., Nagar S. (2023). Process and System Clearances in Pharmacokinetic Models: Our Basic Clearance Concepts Are Correct. Drug Metab. Dispos..

[40] Ghasemitabar H., Movagharnejad K. (2016). Estimation of the Normal Boiling Point of Organic Compounds via a New Group Contribution Method. Fluid Phase Equilib..

[41] Nannoolal Y., Rarey J., Ramjugernath D., Cordes W. (2004). Estimation of Pure Component Properties: Part 1. Estimation of the Normal Boiling Point of Non-Electrolyte Organic Compounds via Group Contributions and Group Interactions. Fluid Phase Equilib..

[42] Mao F., Kong Q., Ni W., Xu X., Ling D., Lu Z., Li J. (2016). Melting Point Distribution Analysis of Globally Approved and Discontinued Drugs: A Research for Improving the Chance of Success of Drug Design and Discovery. ChemistryOpen.

[43] Gadaleta D., Vuković K., Toma C., Lavado G.J., Karmaus A.L., Mansouri K., Kleinstreuer N.C., Benfenati E., Roncaglioni A. (2019). SAR and QSAR Modeling of a Large Collection of LD50 Rat Acute Oral Toxicity Data. J. Cheminform..

[44] Tong G.C., Cornwell W.K., Means G.E. (2004). Reactions of Acrylamide with Glutathione and Serum Albumin. Toxicol. Lett..

[45] Danyaya A.I., Kumar A., Hamza U.A., Yahaya N.L. (2020). Virtual Screening, Molecular Docking, and ADME/T Analysis of Natural Product Library Against Cell Invasion Protein SipB from Salmonella enterica serotype typhi: In Silico Analysis. Acta Sci. Pharm. Sci..

[46] Tripathi S.K., Muttineni R., Singh S.K. (2013). Extra Precision Docking, Free Energy Calculation and Molecular Dynamics Simulation Studies of CDK2 Inhibitors. J. Theor. Biol..

[47] Et-tazy L., Fedeli R., Khibech O., Lamiri A., Challioui A. (2025). Effects of Monoterpene-Based Biostimulants on Chickpea (*Cicer arietinum* L.) Plants: Functional and Molecular Insights. Biology.

[48] Merzouki M., Khibech O., Fraj E., Bouammali H. (2025). Computational engineering of malonate and tetrazole derivatives targeting SARS-CoV-2 main protease: Pharmacokinetics, docking, and molecular dynamics insights to support the sustainable development goals (SDGs), with a bibliometric analysis. Indonesian J. Sci. Technol..

[49] Khan A., Khan S.U., Khan A., Shal B., Rehman S.U., Rehman S.U., Htar T.T., Khan S., Anwar S., Alafnan A. (2022). Anti-Inflammatory and Anti-Rheumatic Potential of Selective Plant Compounds by Targeting TLR-4/AP-1 Signaling: A Comprehensive Molecular Docking and Simulation Approaches. Molecules.

[50] Imran M., Alshrari A.S., Hafiz M.N., Jawad M.M., Khan A., Alanazi F.J., Asdaq S.M.B. (2025). Exploring Therapeutic Paradigm Focusing on Genes, Proteins, and Pathways to Combat Leprosy and Tuberculosis: A Network Medicine and Drug Repurposing Approach. J. Infect. Public Health.

[51] Amala M., Rajamanikandan S., Prabhu D., Surekha K., Jeyakanthan J. (2019). Identification of Anti-Filarial Leads against Aspartate Semialdehyde Dehydrogenase of Wolbachia Endosymbiont of Brugia Malayi: Combined Molecular Docking and Molecular Dynamics Approaches. J. Biomol. Struct. Dyn..

[52] Camargo P.G., da Silva R.B., Zuma A.A., Garden S.J., Albuquerque M.G., Rodrigues C.R., da Silva Lima C.H. (2025). In Silico Evaluation of N-Aryl-1,10-Phenanthroline-2-Amines as Potential Inhibitors of T. Cruzi GP63 Zinc-Metalloprotease by Docking and Molecular Dynamics Simulations. Sci. Rep..

[53] Pires D.E.V., Blundell T.L., Ascher D.B. (2015). PkCSM: Predicting Small-Molecule Pharmacokinetic and Toxicity Properties Using Graph-Based Signatures. J. Med. Chem..

[54] Daina A., Michielin O., Zoete V. (2017). SwissADME: A Free Web Tool to Evaluate Pharmacokinetics, Drug-Likeness and Medicinal Chemistry Friendliness of Small Molecules. Sci. Rep..

[55] Fu L., Shi S., Yi J., Wang N., He Y., Wu Z., Peng J., Deng Y., Wang W., Wu C. (2024). ADMETlab 3.0: An Updated Comprehensive Online ADMET Prediction Platform Enhanced with Broader Coverage, Improved Performance, API Functionality and Decision Support. Nucleic Acids Res..

[56] Banerjee P., Kemmler E., Dunkel M., Preissner R. (2024). ProTox 3.0: A Webserver for the Prediction of Toxicity of Chemicals. Nucleic Acids Res..

[57] Trott O., Olson A.J. (2010). AutoDock Vina: Improving the Speed and Accuracy of Docking with a New Scoring Function, Efficient Optimization, and Multithreading. J. Comput. Chem..

[58] Abraham M.J., Murtola T., Schulz R., Páll S., Smith J.C., Hess B., Lindah E. (2015). Gromacs: High Performance Molecular Simulations through Multi-Level Parallelism from Laptops to Supercomputers. SoftwareX.

